# Genome Sequences of the Novel Porcine Parvovirus 3, Identified in Guangxi Province, China

**DOI:** 10.1128/genomeA.00036-16

**Published:** 2016-03-03

**Authors:** Hui Zhong, Xiangmin Li, Zekai Zhao, Chunjing An, Peng Wan, Mengge Wu, Huanchun Chen, Ping Qian

**Affiliations:** a State Key Laboratory of Agriculture Microbiology, Huazhong Agricultural University, Wuhan, People’s Republic of China; b Laboratory of Animal Virology, College of Veterinary Medicine, Huazhong Agricultural University, Wuhan, People’s Republic of China

## Abstract

Porcine parvovirus 3 is a novel parvovirus that infects pigs. Here, we report two genome sequences of porcine parvovirus 3 strains GX1 and GX2, which are highly prevalent in Guangxi province. It will help in understanding the epidemiology and molecular characteristics of the porcine parvovirus 3.

## GENOME ANNOUNCEMENT

Porcine parvoviruses are small, single-stranded DNA viruses, The virions have a diameter of 18 to 26 nm. Their genomes are about 4.0 to 6.0 kb and contain two main open reading frames (ORFs), ORF1 and ORF2, encoding for nonstructural proteins (NSP) and capsid proteins (Cap), respectively (1–3).

According to the new taxonomy reported by the International Committee on Taxonomy of Viruses (ICTV) in 2011, parvoviruses are classified into two subfamilies based on their host range: the *Parvovirinae*, which infect vertebrates, and the *Densovirinae*, which mainly infect insects and other arthropods (2). Porcine parvovirus 3, which is also known as porcine hokovirus (PHoV) or porcine partetravirus, belongs to the subfamily *Parvovirinae*. It has been identified in many places, including Hong Kong, Romania, and Germany (4–6).

In this study, two porcine parvovirus 3 (PPV3) strains, GX1 and GX2, were identified in tonsil samples from 13-week-old pigs suspected of having porcine circovirus associated disease. The prevalence of PPV3 is 19.5% in this study. Phylogenetic analysis based on the near-full-length genome indicated that the sequences of PPV3 strains GX1 and GX2 were closely related to the isolates from Hong Kong.

The genome was generated with PCR using seven pairs of primers, which were designed using Primer Premier version 5.0 software from alignments of available PPV3 genomes. The PCR products were purified using a TIANgel Midi purification kit (Tiangen). The target DNA fragments were cloned into pMD18-T vector (TaKaRa) and sequenced by TSINGKE Biological Technology (Wuhan, China).

The genome of PPV3 strain GX1 and GX2 is a single-stranded DNA molecule comprising 5,081 bp, with a GC content of 51.5%. It encodes two ORFs, ORF1 and ORF2. ORF1 encodes NS1(636 amino acids [aa]), and ORF2 encodes Cap containing VP1 (925 aa) and VP2 (555 aa). The complete genome of the two strains showed a high nucleotide homology (96.6 to 96.7%) to reference strain PHoV-HK7 (EU200677). When the amino acid sequences of VP2 were analyzed, strain GX1 had two changes (56V → 56T, 252V → 252A), and strain GX2 had three changes (56V → 56T 252V→252 A, 257R → 257G), compared with the reference strain PHoV-HK7. This indicated that PPV3 strains GX1 and GX2 showed a close relationship to the PHoVs and that PPV3 had undergone an evolution in its continuous transmission. These data will provide valuable information about the evolutionary characteristics and genetic diversity of PPV3.

### Nucleotide sequence accession numbers.

The genome sequences of PPV3 strains GX1 and GX2 were deposited in GenBank under accession numbers KU167028 and KU167029.
